# ABO and RhD Blood Groups in Epilepsy: A Comprehensive Case–Control Analysis

**DOI:** 10.3390/jcm15186949

**Published:** 2026-09-08

**Authors:** Okan Sokmen, Osman Korucu

**Affiliations:** Department of Neurology, Atatürk Sanatorium Research and Training Hospital, Ankara 06280, Türkiye

**Keywords:** epilepsy, ABO blood group, RhD factor, blood group distribution, case–control study, epidemiology

## Abstract

**Background:** Although ABO blood groups have been implicated in several vascular and neurological disorders, evidence regarding their association with epilepsy remains limited and inconclusive. We performed the largest and most comprehensive case–control evaluation to date to determine whether ABO/RhD blood groups are associated with epilepsy. **Methods:** We conducted a retrospective hospital-based case–control study including 806 adults with epilepsy and 14,032 controls from the same institutional population between January 2015 and January 2026. Epilepsy diagnoses were confirmed independently by two neurologists according to International League Against Epilepsy criteria. Associations between epilepsy and ABO/RhD blood groups were evaluated using a comprehensive analytical framework incorporating multivariable-adjusted logistic regression, binary blood group comparisons, age- and sex-matched sensitivity analyses, and epilepsy subtype-specific analyses. **Results:** No significant association was identified between epilepsy and ABO/RhD blood groups across any analytical approach. Overall ABO blood group (*p* = 0.777) and RhD status (*p* = 0.611) distributions were comparable between patients with epilepsy and controls. Binary analyses showed no significant associations for non-O versus O (OR 1.05, 95% CI 0.90–1.22; *p* = 0.535), non-A versus A (OR 0.93, 95% CI 0.80–1.06; *p* = 0.297), non-B versus B (OR 1.03, 95% CI 0.85–1.25; *p* = 0.748), non-AB versus AB (OR 1.05, 95% CI 0.81–1.34; *p* = 0.723), or RhD-negative versus RhD-positive status (OR 1.06, 95% CI 0.85–1.31; *p* = 0.611). These findings remained unchanged after multivariable-adjusted logistic regression, 1:4 age- and sex-matched sensitivity analyses (806 patients with epilepsy and 3224 matched controls; ABO *p* = 0.774, RhD *p* = 0.740), and comparisons between focal and generalized epilepsy subtypes (ABO *p* = 0.531; RhD *p* = 0.995). **Conclusions:** In this large hospital-based case–control study, no significant association was identified between epilepsy and ABO/RhD blood groups. However, the hospital-based control population and the availability of blood group data should be considered when interpreting these findings.

## 1. Introduction

Epilepsy affects approximately 52 million people worldwide and remains one of the most common serious neurological disorders across all age groups [1,2]. Although major advances have been made in neuroimaging, electrophysiology, genetics, and molecular neuroscience, the mechanisms underlying epileptogenesis are still not fully understood. In recent years, increasing attention has focused on the potential contribution of vascular, inflammatory, hematological, and immunological factors to seizure susceptibility.

The ABO blood group system is one of the most extensively studied hereditary blood group classifications in medicine. Beyond its role in transfusion practice, ABO blood groups have been associated with various cardiovascular, thrombotic, inflammatory, metabolic, and neurological disorders [3,4]. Previous studies have shown that non-O blood groups may be associated with increased thrombotic tendency through higher circulating levels of von Willebrand factor and factor VIII [4]. In addition, phenome-wide association studies have demonstrated significant associations between ABO blood groups and multiple systemic disease phenotypes [3].

Despite growing interest in the clinical implications of ABO blood groups, studies evaluating their possible association with epilepsy remain scarce. Earlier studies suggested that certain ABO blood groups might be overrepresented among patients with epilepsy, raising the possibility of a biological association between blood group antigens and seizure susceptibility [5]. More recent studies have investigated the distribution of ABO and RhD blood groups in epilepsy populations; however, the available evidence remains limited, inconsistent, and largely based on relatively small single-center cohorts [6]. Consequently, the potential role of ABO and RhD blood groups as susceptibility factors for epilepsy remains uncertain.

Therefore, the present study aimed to investigate the distribution of ABO and RhD blood groups in patients with epilepsy and compare these findings with a large hospital-based control population. In addition, binary blood group comparisons, multivariable-adjusted analyses, matched sensitivity analyses, and epilepsy subtype analyses were performed to further evaluate potential associations between blood groups and epilepsy.

## 2. Methods

### 2.1. Study Design and Setting

This retrospective, observational, case–control study was conducted at a tertiary care training and research hospital in Türkiye. Electronic medical records of adult patients followed with a diagnosis of epilepsy between January 2015 and January 2026 were retrospectively reviewed.

The diagnosis of epilepsy was confirmed by two board-certified neurologists according to International League Against Epilepsy (ILAE) criteria [7,8], based on clinical history, neurological examination, electroencephalographic findings and neuroimaging findings when available, and follow-up information documented in the electronic medical records.

### 2.2. Study Population

Patients aged 18 years and older with a confirmed diagnosis of epilepsy according to ILAE criteria, followed in the neurology outpatient clinic between January 2015 and January 2026, and with available ABO/RhD blood group data and sufficient clinical records were included in the epilepsy group. All patients who met these eligibility criteria and had documented ABO/RhD blood group data were included.

The control group consisted of individuals without epilepsy who were identified from the same hospital database during the study period. Controls were drawn from the general neurology outpatient clinic rather than from disease-specific subspecialty clinics. Individuals with epilepsy, acute symptomatic seizures, psychogenic nonepileptic seizures, or missing ABO/RhD blood group data were excluded. Patients with cerebrovascular disease, including ischemic or hemorrhagic stroke and transient ischemic attack, were also excluded regardless of their presenting complaint. This included patients with a previously known cerebrovascular diagnosis as well as those diagnosed with cerebrovascular disease after presenting with an unrelated or nonspecific complaint. Patients with other major vascular conditions, including coronary artery disease, pulmonary thromboembolism, and peripheral arterial disease, were also excluded, even when the reason for neurological evaluation was unrelated to the vascular condition.

The control group included patients with a wide range of reasons for neurological evaluation. Common presentations included headache, dizziness or balance complaints, numbness or paresthesia, neck, back or hip pain, memory complaints, tremor, syncope or presyncope, sleep-related complaints, and psychiatric or functional symptoms. The group also included individuals without a known chronic neurological disorder who attended for routine or administrative evaluations, such as driver’s license, school or dormitory admission, sports participation, course enrollment, or assessment of decision-making or legal capacity. Some individuals attended the neurology clinic for nonspecific or unrelated reasons. Presenting complaints and reasons for evaluation were grouped for descriptive purposes and are presented in Supplementary Table S1. These categories reflect the reason for presentation rather than systematically established final neurological diagnoses.

### 2.3. Data Collection and Variables

All study data were obtained from the hospital information management system and laboratory database. No additional laboratory tests, interventions, or blood sampling procedures were performed for the purpose of this study. The recorded variables included age, sex, presence or absence of epilepsy, epilepsy type, ABO blood group, and RhD status. All data were collected using a standardized data collection form and anonymized before analysis.

The primary outcome of the study was the comparison of ABO blood group distribution between patients with epilepsy and controls. Secondary analyses included comparisons of RhD status, binary blood group distributions (A/non-A, O/non-O, B/non-B, and AB/non-AB), and ABO/RhD blood group distributions according to epilepsy subtype.

### 2.4. Classification of Epilepsy

Patients with acute symptomatic seizures, psychogenic nonepileptic seizures, syncope, or single unprovoked seizures without a diagnosis of epilepsy were excluded.

Seizure types and epilepsy classification were determined according to the ILAE classification system [7]. When sufficient clinical information was available, patients were classified as having focal epilepsy or generalized epilepsy based on clinical semiology, electroencephalographic findings, neuroimaging findings, and neurologist documentation in medical records. Cases that could not be clearly categorized according to available electroclinical data were not included in subtype analyses.

For the etiological analysis, epilepsy etiology was classified as genetic or non-genetic based on the available clinical, electroencephalographic, neuroimaging, and medical record information; cases in which etiology could not be reliably determined were categorized as undetermined/unclassifiable and excluded from the etiological subgroup analysis.

### 2.5. ABO and RhD Blood Group Assessment

ABO and RhD blood group data were obtained from the hospital laboratory information system. Blood group analyses had been previously performed as part of routine clinical care using standard serological gel column agglutination methods in the institutional transfusion laboratory. No additional blood sampling or laboratory testing was performed for the purpose of this study.

### 2.6. Statistical Analysis

Statistical analyses were performed using IBM SPSS Statistics software (Version 25.0; IBM Corp., Armonk, NY, USA) and Jamovi software (Version 2.6; The Jamovi Project, Sydney, Australia). Continuous variables were expressed as mean ± standard deviation or median (interquartile range) according to data distribution, while categorical variables were presented as numbers and percentages. Comparisons between groups were performed using Student’s *t*-test or Mann–Whitney U test for continuous variables and chi-square test or Fisher’s exact test for categorical variables, as appropriate.

ABO blood groups were analyzed both as four-category variables (A, B, AB, and O) and as binary comparisons, including A versus non-A, O versus non-O, B versus non-B, and AB versus non-AB. RhD status was analyzed as RhD-positive versus RhD-negative. Odds ratios (ORs) and 95% confidence intervals (95% CIs) were calculated to evaluate the association between blood groups and epilepsy. Separate multivariable logistic regression models were fitted to evaluate the association between ABO/RhD blood groups and epilepsy after adjustment for age and sex, with epilepsy status as the dependent variable in each model. The primary model included the four-category ABO variable (blood group A as the reference category), age, and sex as independent variables. Separate models were then constructed for each binary blood-group comparison (non-O vs. O, non-A vs. A, non-B vs. B, non-AB vs. AB, and RhD− vs. RhD+), each adjusted for age and sex. The four-category ABO variable and the binary blood-group comparisons were not entered into the same model because they represent alternative codings of the same variable and are therefore inherently collinear. Model fit was assessed using the Hosmer–Lemeshow goodness-of-fit test and Nagelkerke R^2^, and multicollinearity among the independent variables was evaluated using variance inflation factors (VIFs). All models were fitted using the forced-entry (Enter) method, with all covariates entered simultaneously. No interaction terms were included in any model.

To further address potential demographic imbalance between groups, an additional age- and sex-matched sensitivity analysis was performed using 1:4 nearest-neighbor matching without replacement, and ABO/RhD blood group distributions were subsequently reanalyzed in the matched cohort. Additional subgroup analyses were performed according to epilepsy subtype (focal vs. generalized) and underlying etiology (genetic vs. non-genetic), comparing demographic characteristics, ABO blood group distributions, binary blood group comparisons, and RhD status between each pair of subgroups; patients with insufficient electroclinical information for subtype classification, or with undetermined/unclassifiable etiology, were excluded from the respective analysis. Patients with missing ABO/RhD blood group data or insufficient clinical records were excluded from the analysis, and no imputation method was applied for missing data. A two-sided *p*-value < 0.05 was considered statistically significant. 

To quantify the statistical power of our case–control comparisons, we performed a post hoc minimum detectable effect size (MDES) analysis. For each binary blood-group comparison (non-O vs. O, non-A vs. A, non-B vs. B, non-AB vs. AB, and RhD− vs. RhD+) and for the overall ABO/RhD distribution comparisons, we calculated the smallest odds ratio (OR), or Cohen’s w for the chi-square-based comparisons, that the achieved sample sizes would provide 80% power to detect at a two-sided α of 0.05, using the observed control-group prevalence as the reference proportion. This analysis was repeated separately for the primary case–control cohort and the age- and sex-matched sensitivity cohort. Power calculations were performed using the statsmodels package (version 0.14) in Python.

### 2.7. Ethical Considerations

The study was approved by the Scientific Research Ethics Committee of Atatürk Sanatorium Research and Training Hospital (Decision No: 2024/579, dated 20 May 2026). The study was conducted in accordance with the principles of the Declaration of Helsinki. Owing to the retrospective nature of the study and the use of anonymized data, informed consent was waived by the ethics committee.

**Figure 1:**
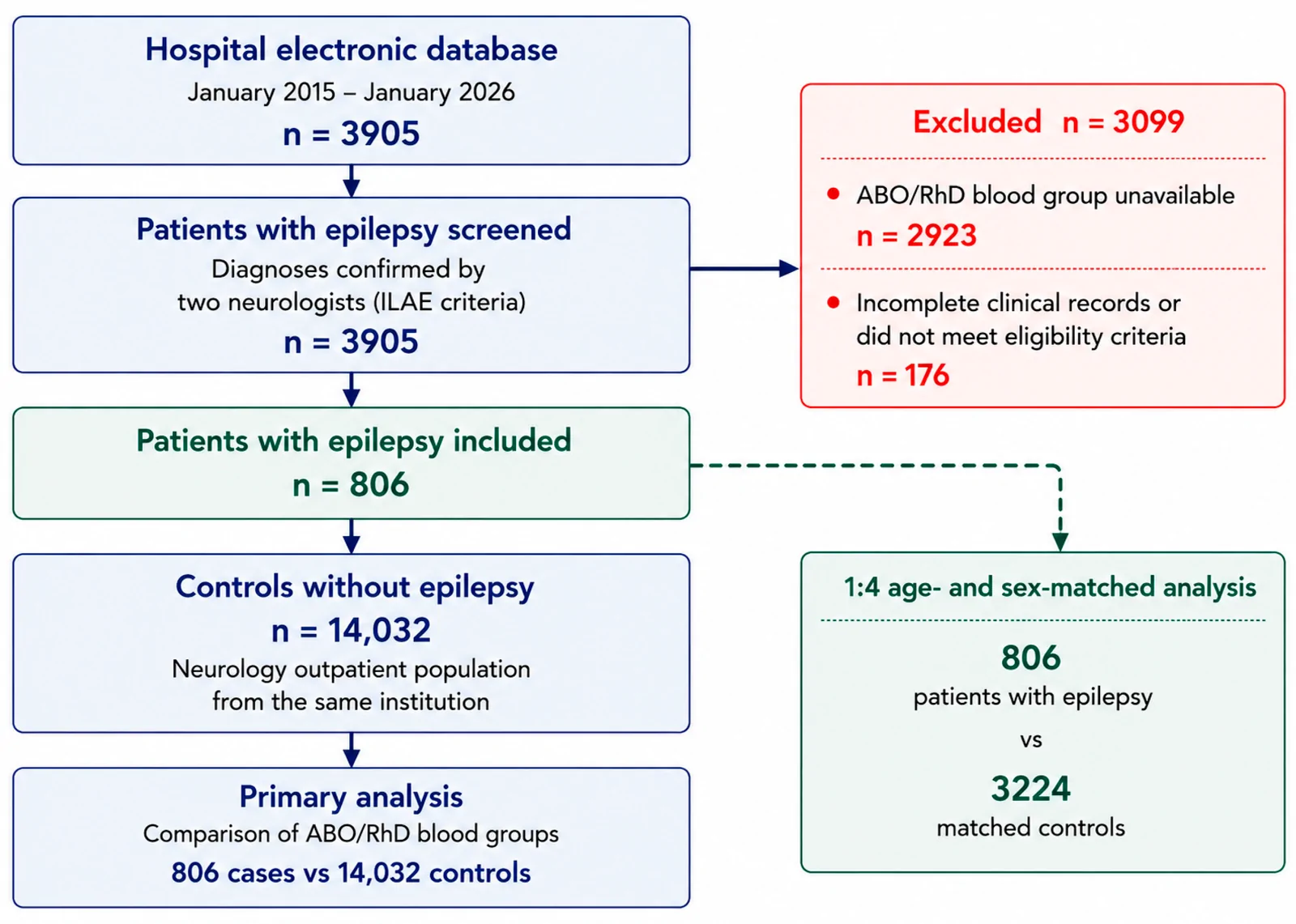
Flowchart of study population selection and analytical strategy. A total of 3905 patients with an epilepsy diagnosis were screened. Following exclusion of patients with unavailable ABO/RhD blood group data, incomplete clinical records, or failure to meet the study eligibility criteria, 806 patients were included in the final analysis. The control group comprised 14,032 individuals without epilepsy. A 1:4 age- and sex-matched sensitivity analysis was also performed.

The overall study workflow is summarized in Figure 1.

## 3. Results

### 3.1. Demographic Characteristics and Detailed ABO/RhD Blood Group Distribution

A total of 3905 patients with an epilepsy diagnosis were identified from the hospital electronic database. Of these, 2923 had no documented ABO/RhD blood group result and therefore could not be evaluated for the primary exposure of interest. A further 176 patients had incomplete clinical records or did not meet the predefined eligibility criteria. The remaining 806 eligible patients with available ABO/RhD data constituted the final epilepsy cohort (Figure 1). A total of 14,032 individuals without epilepsy were included as controls. The control group included individuals with a broad range of reasons for neurological evaluation. The most frequent categories were headache (18.7%), musculoskeletal or pain complaints (18.3%), sensory complaints (14.2%), dizziness or balance complaints (14.1%), and routine or administrative evaluations (9.8%). The complete distribution of presenting complaints and reasons for evaluation is provided in Supplementary Table S1.

The mean age was 52.8 ± 19.7 years in the epilepsy group and 58.3 ± 19.3 years in the control group. The epilepsy group was significantly younger than controls (*p* < 0.001). Male patients accounted for 46.5% (n = 375) of the epilepsy group and 37.0% (n = 5188) of the control group, whereas female patients accounted for 53.5% (n = 431) and 63.0% (n = 8844), respectively.

The distributions of ABO blood groups and RhD status are presented separately in Table 1. For descriptive purposes, the combined ABO/RhD distribution was also examined. An RhD-positive was the most frequent phenotype in both the epilepsy and control groups (38.3% vs. 35.9%), followed by O RhD-positive (27.4% vs. 28.2%), while AB RhD-negative was the least frequent (1.5% vs. 1.3%).

### 3.2. Distribution and Binary Comparison of ABO/RhD Blood Groups

The distribution of ABO blood groups was similar between the epilepsy and control groups (*p* = 0.777). Blood group A was the most common ABO type in both groups (43.1% vs. 41.2%), followed by O (31.1% vs. 32.2%), B (17.0% vs. 17.4%), and AB (8.8% vs. 9.2%) (Table 1).

The non-O versus O comparison showed no significant difference between the groups (OR: 1.05, 95% CI: 0.90–1.22, *p* = 0.535). Similarly, no significant associations were observed for non-A versus A (OR: 0.93, 95% CI: 0.80–1.06, *p* = 0.297), non-B versus B (OR: 1.03, 95% CI: 0.85–1.25, *p* = 0.748), or non-AB versus AB comparisons (OR: 1.05, 95% CI: 0.81–1.34, *p* = 0.723) (Table 2).

RhD blood group distribution was also comparable between the epilepsy and control groups (*p* = 0.611). RhD positivity was observed in 87.7% of epilepsy patients and 87.1% of controls (Table 1). No significant association was identified between RhD status and epilepsy (OR: 1.06, 95% CI: 0.85–1.31, *p* = 0.611) (Table 2).

**Figure 2:**
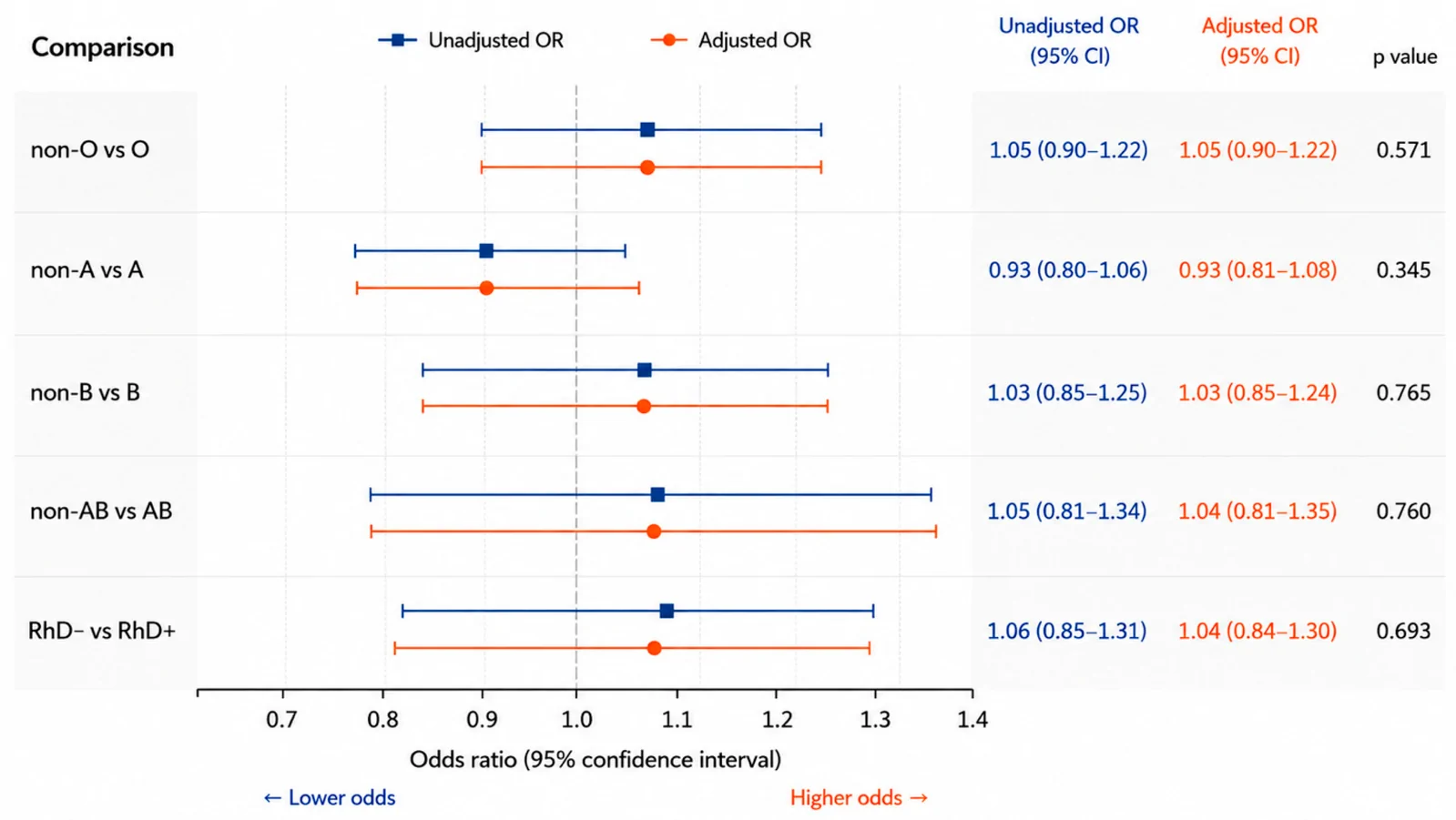
Unadjusted and adjusted odds ratios for the association between ABO/RhD blood groups and epilepsy. Forest plot showing unadjusted (blue squares) and age- and sex-adjusted (orange circles) odds ratios with 95% confidence intervals for binary ABO/RhD blood group comparisons. The vertical dashed line indicates an odds ratio of 1.0 (no association). *p* values displayed correspond to the age- and sex-adjusted logistic regression analyses.

Because age and sex differed between the epilepsy and control groups, additional multivariable logistic regression analyses adjusted for these variables were performed (Table 2). Increasing age was independently associated with lower odds of belonging to the epilepsy group (aOR: 0.981, 95% CI: 0.977–0.985, *p* < 0.001), whereas female sex was associated with lower odds of belonging to the epilepsy group compared with male sex (aOR: 0.545, 95% CI: 0.469–0.634, *p* < 0.001). After adjustment, no significant associations were observed between epilepsy and blood group O versus A (adjusted OR [aOR]: 1.07, 95% CI: 0.91–1.27, *p* = 0.409), blood group B versus A (aOR: 0.94, 95% CI: 0.76–1.15, *p* = 0.539), or blood group AB versus A (aOR: 0.93, 95% CI: 0.72–1.21, *p* = 0.592). Likewise, no significant associations were identified for non-O versus O (aOR: 1.05, 95% CI: 0.90–1.22, *p* = 0.571), non-A versus A (aOR: 0.93, 95% CI: 0.81–1.08, *p* = 0.345), non-B versus B (aOR: 1.03, 95% CI: 0.85–1.24, *p* = 0.765), non-AB versus AB (aOR: 1.04, 95% CI: 0.81–1.35, *p* = 0.760), or RhD− versus RhD+ status (aOR: 1.04, 95% CI: 0.84–1.30, *p* = 0.693) (Table 2). The adjusted and unadjusted odds ratios are illustrated in Figure 2. Goodness-of-fit and multicollinearity diagnostics for each model, including Nagelkerke R^2^, the Hosmer–Lemeshow test, and variance inflation factors, are presented in Supplementary Table S2. All variance inflation factors were below 1.3, indicating no evidence of problematic multicollinearity among the predictors. Nagelkerke R^2^ values were approximately 0.024 across the models, indicating limited overall explanatory capacity. The Hosmer–Lemeshow test was statistically significant in several models, indicating potential calibration limitations. These regression models were used to obtain age- and sex-adjusted estimates of the prespecified associations between ABO/RhD blood groups and epilepsy status and were not intended as predictive models.

We also performed a 1:4 age- and sex-matched sensitivity analysis to confirm the primary findings in a matched cohort of 806 patients with epilepsy and 3224 controls (Table 3). After matching, age and sex distributions were comparable between groups. Consistent with the primary analysis, no significant difference was observed in overall ABO blood group distribution (*p* = 0.774) or RhD status (*p* = 0.740) between the epilepsy and matched control groups (Table 3).

The primary case–control comparison (806 vs. 14,032) provided 80% power to detect odds ratios as small as approximately 0.72–0.82 (protective) or 1.22–1.46 (risk), depending on the baseline prevalence of each blood group (Supplementary Table S3). The observed odds ratios for all comparisons (range 0.93–1.06) were substantially closer to the null value than the corresponding minimum detectable effect sizes. Similarly, the study had 80% power to detect a Cohen’s *w* of 0.023–0.027 for the overall ABO and RhD distribution comparisons, whereas the observed *w* values were 0.004–0.009.

### 3.3. ABO/RhD Blood Groups According to Epilepsy Type

Among 806 patients with epilepsy, 470 (58.3%) were classified as having focal epilepsy, 138 (17.1%) as generalized epilepsy, and 198 (24.6%) as unclassified epilepsy. After exclusion of unclassified cases, subtype analyses were performed in 608 patients, including 470 (77.3%) with focal epilepsy and 138 (22.7%) with generalized epilepsy. Patients with generalized epilepsy were significantly younger than those with focal epilepsy (38.6 ± 17.2 vs. 54.6 ± 18.4 years, *p* < 0.001), whereas sex distribution was similar between the groups (*p* = 0.212). The overall distribution of ABO blood groups did not differ significantly between focal and generalized epilepsy (*p* = 0.531). Similarly, no significant differences were observed in analyses comparing A versus non-A (*p* = 0.718), O versus non-O (*p* = 0.819), B versus non-B (*p* = 0.417), or AB versus non-AB blood groups (*p* = 0.191). RhD distribution was also comparable between the groups, with no significant association between RhD status and epilepsy subtype (*p* = 0.995) (Table 4).

### 3.4. ABO/RhD Blood Groups According to Genetic Etiology of Epilepsy

Of the 806 patients with epilepsy, etiology could be classified as genetic in 154 (19.1%) and non-genetic in 448 (55.6%), while 204 (25.3%) had undetermined or unclassifiable etiology and were excluded from this subanalysis. After exclusion of undetermined cases, comparative analyses were performed in 602 patients, including 154 (25.6%) with genetic epilepsy and 448 (74.4%) with non-genetic epilepsy. Patients with genetic epilepsy were significantly younger than those with non-genetic epilepsy (43.9 ± 20.4 vs. 54.6 ± 18.4 years, *p* < 0.001), whereas sex distribution was similar between the groups (*p* = 0.802). The overall distribution of ABO blood groups did not differ significantly between genetic and non-genetic epilepsy (*p* = 0.678). Similarly, no significant differences were observed in analyses comparing A versus non-A (*p* = 0.511), O versus non-O (*p* = 0.713), B versus non-B (*p* = 0.682), or AB versus non-AB blood groups (*p* = 0.285). RhD status distribution was also comparable between the groups (*p* = 0.813) (Table 5). Given the significant age difference between the genetic and non-genetic epilepsy groups, additional logistic regression analyses were performed to evaluate the associations between ABO/RhD blood groups and genetic epilepsy status after adjustment for age and sex. None of the ABO/RhD comparisons reached statistical significance (all *p* > 0.05; Supplementary Table S4).

## 4. Discussion

In this large hospital-based case–control study, patients with epilepsy were compared to a control group consisting of general neurology outpatients without epilepsy from the same institution. No significant association was identified between epilepsy and ABO blood group or RhD distribution. Blood group A was the most common type in both groups, followed by O, B, and AB, consistent with the reported ABO blood group distribution in the Turkish population [9,10]. RhD positivity rates were comparable between groups, and no meaningful association was identified between RhD status and epilepsy. The findings were consistent after multivariable adjustment and age- and sex-matched sensitivity analyses. However, these analyses address measured demographic differences and cannot eliminate potential selection related to the composition of the hospital-based control group or the availability of blood group data. Furthermore, subtype analyses demonstrated no significant differences in ABO blood group distribution or RhD status between focal and generalized epilepsy, suggesting that blood group-related susceptibility does not appear to differ across major epilepsy subtypes. Focal epilepsy accounted for approximately three quarters of cases in our cohort, which is comparable to the proportions reported in other adult hospital-based series [6,11]. However, direct comparisons between studies should be made cautiously, as differences in study populations, referral patterns, and the use of the current ILAE epilepsy classification may influence the reported distribution of epilepsy subtypes.

Similarly, no significant differences in ABO/RhD blood group distribution were observed between patients with genetic and non-genetic epilepsy, and the findings remained non-significant after adjustment for age and sex. Etiology is an important dimension of epilepsy classification, with the ILAE recognizing structural, genetic, infectious, metabolic, immune, and unknown etiological categories [12]. Etiological heterogeneity could theoretically obscure a blood group association confined to a specific pathogenic subgroup. However, the absence of a significant difference between the genetic and non-genetic groups in our cohort provides no evidence that the overall null association was driven primarily by genetic etiology. This finding should nevertheless be interpreted cautiously, as our retrospective data permitted only a broad genetic versus non-genetic classification and 25.3% of patients had an undetermined or unclassifiable etiology. Therefore, potential associations with specific etiological categories cannot be excluded.

Studies directly evaluating ABO/RhD blood group distribution in epilepsy remain scarce. Historically, one of the earliest systematic investigations of ABO blood groups in neurological and psychiatric disorders was published by Thomas and Hewitt in 1939 [13], who found no evidence of an association between ABO blood groups and mental illness. A subsequent report published in 1972 suggested possible overrepresentation of blood groups A and AB among women with epilepsy [5]; however, the study included a relatively small and sex-restricted cohort, substantially limiting the generalizability of its findings. The study most comparable to ours was conducted by Asadi-Pooya et al. in Iran in 2022 [6]. In that study, which included 390 patients with epilepsy and 7672 controls, no significant differences in ABO or RhD blood group distribution were identified between groups. Our findings are consistent with those results and further support the absence of a significant association between ABO/RhD blood groups and epilepsy. The present study extends these previous findings by evaluating a larger epilepsy cohort and control population and by incorporating multivariable-adjusted logistic regression, age- and sex-matched sensitivity analyses, and epilepsy subtype-specific comparisons. The consistency of findings across all these approaches together with adjusted odds ratios close to unity and confidence intervals centered around the null value, suggests that any association between ABO/RhD blood groups and epilepsy, if present, is likely to be small. A post hoc minimum detectable effect size analysis indicated that the study had 80% power to detect odds ratios as small as approximately 0.72–1.46 across the binary blood-group comparisons (Supplementary Table S3). As the observed odds ratios (0.93–1.06) were considerably closer to the null value than these minimum detectable effect sizes, the null findings are unlikely to reflect inadequate statistical power.

The available evidence has not shown a consistent association between ABO/RhD blood groups and epilepsy. Unlike thrombotic or cardiovascular disorders, in which ABO-related biological effects appear relatively strong and reproducible, epilepsy is a highly heterogeneous neurological condition shaped by complex interactions among genetic, structural, inflammatory, developmental, and environmental factors. Any potential contribution of ABO-related pathways to epileptogenesis may therefore be too modest or biologically indirect to produce a consistent epidemiological signal in broad clinical cohorts. The marked clinical and biological heterogeneity of epilepsy may further complicate the detection of weak population-level associations involving genetically determined blood group antigens.

Interestingly, a large phenome-wide association study using UK Biobank data identified significant associations between ABO-related genetic variants and several cardiovascular, thrombotic, and hematological phenotypes, but not with neurological diseases [3]. Although indirect, these genomic findings are broadly consistent with our clinical results and further support the absence of a major population-level association between ABO blood groups and epilepsy. Nevertheless, several biological mechanisms potentially linking ABO blood groups and epileptogenesis have been proposed. ABO antigens are expressed not only on erythrocytes but also on vascular endothelium and neural tissues [6,14], while RhD-related proteins have additionally been detected in nonerythroid tissues including the brain [6,15]. Non-O blood groups are associated with higher circulating von Willebrand factor levels, which may influence blood–brain barrier integrity and neurovascular responses during seizures [4,16,17,18]. In addition, ABO-related differences in inflammatory signaling and gut microbiota composition have been proposed as potential modulators of neuroinflammatory processes involved in epilepsy [19,20,21,22]. However, these proposed mechanisms remain largely hypothetical, as direct experimental evidence linking ABO/RhD blood groups to epileptogenesis is currently lacking. The absence of a significant association in both the primary and matched analyses further suggests that any contribution of these pathways is likely to be limited.

The present study has several strengths. First, it included a substantially larger epilepsy cohort and control population than previous studies investigating ABO/RhD blood groups in epilepsy. Second, both groups were derived from the same institutional population, which helped limit regional demographic differences between groups. In addition, epilepsy diagnoses were confirmed by two neurologists according to ILAE criteria, increasing the reliability of case classification. Age- and sex-matched sensitivity analyses provided an additional assessment of the observed associations and yielded findings consistent with the primary analysis. Given the limited number of population-based studies investigating ABO/RhD blood group distribution in epilepsy, the relatively large cohort size of the present study provides additional value to the currently sparse literature. Another strength is the clinically characterized epilepsy cohort, which allowed subtype analyses and assessment of potential differences between focal and generalized epilepsy.

Several limitations should be considered when interpreting the findings of this study. First, the retrospective design relied on electronic medical records, which may have resulted in incomplete clinical data in some patients. Second, this was a single-center study, which may limit the generalizability of the findings to other populations. ABO/RhD blood group data were unavailable for 2923 of the 3905 initially identified patients with epilepsy. All otherwise eligible patients with available ABO/RhD data were included, and no investigator-directed sampling based on demographic or clinical characteristics was performed. Nevertheless, because the availability of ABO/RhD data may not have been random, selection related to blood group data availability cannot be completely excluded. 

In addition, the control group consisted of hospital-based general neurology outpatients rather than healthy community controls. Although the available presenting complaints and reasons for evaluation allowed us to characterize the clinical composition of the control group, final neurological diagnoses were not systematically recorded for all control participants, and reliable diagnosis-specific analyses could not be performed. Patients with documented cerebrovascular disease and major vascular comorbidities were excluded, including those in whom cerebrovascular disease was identified during the evaluation of an initially unrelated complaint. Nevertheless, the hospital-based nature and clinical composition of the control group may have influenced the observed ABO/RhD distribution, and residual selection or collider bias cannot be completely excluded.

We were also unable to perform sufficiently detailed analyses across individual etiological or syndrome-specific categories. Therefore, although the genetic versus non-genetic analysis did not identify a significant association, potential blood group differences within specific etiological categories or epilepsy syndrome cannot be excluded. Additional multivariable-adjusted and age- and sex-matched sensitivity analyses yielded findings consistent with the primary analysis; however, these analyses cannot eliminate potential bias related to the clinical composition of the hospital-based control group. The multivariable models also showed limited overall explanatory capacity, and several models showed potential calibration limitations. The adjusted analyses were intended to estimate associations rather than to predict epilepsy status. 

## 5. Conclusions

In conclusion, this large hospital-based case–control study found no significant association between epilepsy and ABO/RhD blood group distribution. The findings remained consistent after multivariable adjustment and matched sensitivity analyses, although these analyses do not eliminate the limitations related to the hospital-based control population and blood group data availability. Although several biological mechanisms linking blood group antigens and epileptogenesis have been proposed, no significant association was detected in the present study. Larger multicenter studies incorporating etiological, syndrome-specific, and epilepsy subtype-specific classifications may further clarify this relationship.

## Figures and Tables

**Table 1:** Demographic and clinical characteristics of the study population.

Variable	Epilepsy Group (n = 806)	Control Group (n = 14,032)	*p* Value
Age, mean ± SD years	52.8 ± 19.7	58.3 ± 19.3	**<0.001**
Male sex, n (%)	375 (46.5)	5188 (37.0)	**<0.001**
Female sex, n (%)	431 (53.5)	8844 (63.0)	
**ABO blood group, n (%)**			0.777
A	347 (43.1)	5780 (41.2)	
B	137 (17.0)	2447 (17.4)	
AB	71 (8.8)	1288 (9.2)	
O	251 (31.1)	4517 (32.2)	
**RhD status, n (%)**			0.611
RhD+	707 (87.7)	12,222 (87.1)	
RhD−	99 (12.3)	1810 (12.9)	

Data are presented as mean ± SD or n (%). Continuous variables were compared using the independent samples *t*-test, and categorical variables using the χ^2^ test.

**Table 2:** Unadjusted and age- and sex-adjusted associations between ABO/RhD blood groups and epilepsy.

Variable	Unadjusted OR (95% CI)	*p* Value	Adjusted OR (95% CI)	*p* Value
Blood group O vs. A	—	—	1.07 (0.91–1.27)	0.409
Blood group B vs. A	—	—	0.94 (0.76–1.15)	0.539
Blood group AB vs. A	—	—	0.93 (0.72–1.21)	0.592
non-O vs. O	1.05 (0.90–1.22)	0.535	1.05 (0.90–1.22)	0.571
non-A vs. A	0.93 (0.80–1.06)	0.297	0.93 (0.81–1.08)	0.345
non-B vs. B	1.03 (0.85–1.25)	0.748	1.03 (0.85–1.24)	0.765
non-AB vs. AB	1.05 (0.81–1.34)	0.723	1.04 (0.81–1.35)	0.760
RhD− vs. RhD+	1.06 (0.85–1.31)	0.611	1.04 (0.84–1.30)	0.693

Adjusted odds ratios were obtained from separate multivariable logistic regression models including age and sex as covariates, with epilepsy status as the dependent variable. Blood group A and RhD-positive status served as the reference categories for categorical analyses. The four-category ABO comparison, each binary ABO comparison, and the RhD comparison were analyzed in separate models because these variables represent alternative codings of the same blood group information and are therefore collinear. For binary comparisons, the first-listed group was compared with the second-listed reference group. OR, odds ratio; CI, confidence interval.

**Table 3:** Age- and sex-matched sensitivity analysis of ABO/RhD blood groups between epilepsy and control groups.

Variable	Epilepsy (n = 806)	Matched Control (n = 3224)	*p* Value	Sensitivity Analysis OR (95% CI)	*p* Value
Age, mean ± SD, years	52.8 ± 19.7	52.8 ± 19.7	0.999	—	—
Male sex, n (%)	375 (46.5)	1500 (46.5)	1.000	—	—
Female sex, n (%)	431 (53.5)	1724 (53.5)		—	—
**ABO blood group, overall**			0.774	—	—
A	347 (43.1)	1331 (41.3)	—	—	—
B	137 (17.0)	589 (18.3)	—	—	—
AB	71 (8.8)	291 (9.0)	—	—	—
O	251 (31.1)	1013 (31.4)	—	—	—
non-O vs. O	—	—	—	1.01 (0.86–1.20)	0.878
non-A vs. A	—	—	—	0.93 (0.80–1.09)	0.362
non-B vs. B	—	—	—	1.09 (0.89–1.34)	0.401
non-AB vs. AB	—	—	—	1.03 (0.78–1.35)	0.847
**RhD status, overall**			0.740	—	—
RhD-positive	707 (87.7)	2814 (87.3)	—	—	—
RhD-negative	99 (12.3)	410 (12.7)	—	—	—
RhD− vs. RhD+	—	—	—	1.04 (0.82–1.32)	0.740

Data are presented as mean ± SD or n (%). Cases were matched to controls in a 1:4 ratio on age and sex. Continuous variables were compared using the independent samples *t*-test, and categorical variables using the χ^2^ test. Adjusted ORs were obtained from separate multivariable logistic regression models including age and sex as covariates. Each binary blood-group comparison (non-O vs. O, non-A vs. A, non-B vs. B, non-AB vs. AB, and RhD− vs. RhD+) was analyzed in a separate model.

**Table 4:** Demographic Characteristics and ABO/RhD Blood Group Distribution in Patients with Focal and Generalized Epilepsy.

Variable	Focal (n = 470)	Generalized (n = 138)	*p*
Age, years	54.6 ± 18.4	38.6 ± 17.2	<0.001
Male sex, n (%)	219 (46.6)	56 (40.6)	0.212
Female sex, n (%)	251 (53.4)	82 (59.4)	
**ABO blood group, n (%)**			0.531 *
A	203 (43.2)	62 (44.9)	
B	78 (16.6)	27 (19.6)	
AB	48 (10.2)	9 (6.5)	
O	141 (30.0)	40 (29.0)	
**Binary comparisons**			
A vs. non-A, n (%)	203 (43.2)/267 (56.8)	62 (44.9) vs. 76 (55.1)	0.718
O vs. non-O, n (%)	141 (30.0) vs. 329 (70.0)	40 (29.0) vs. 98 (71.0)	0.819
B vs. non-B, n (%)	78 (16.6) vs. 392 (83.4)	27 (19.6) vs. 111 (80.4)	0.417
AB vs. non-AB, n (%)	48 (10.2) vs. 422 (89.8)	9 (6.5) vs. 129 (93.5)	0.191
**RhD+ vs. RhD−, n (%)**	412 (87.7) vs. 58 (12.3)	121 (87.7) vs. 17 (12.3)	0.995

* Overall comparison of ABO blood group distribution (4 × 2 chi-square test).

**Table 5:** Demographic Characteristics and ABO/RhD Blood Group Distribution in Patients with Genetic versus Non-Genetic Epilepsy.

Variable	Genetic (n = 154)	Non-Genetic (n = 448)	*p*
Age, years	43.9 ± 20.4	54.6 ± 18.4	<0.001
Male sex, n (%)	68 (44.2)	205 (45.8)	0.802
Female sex, n (%)	86 (55.8)	243 (54.2)	
**ABO blood group, n (%)**			0.678 *
A	70 (45.5)	190 (42.4)	
B	28 (18.2)	75 (16.7)	
AB	11 (7.1)	45 (10.0)	
O	45 (29.2)	138 (30.8)	
**Binary comparisons**			
A vs. non-A, n (%)	70 (45.5) vs. 84 (54.5)	190 (42.4) vs. 258 (57.6)	0.511
O vs. non-O, n (%)	45 (29.2) vs. 109 (70.8)	138 (30.8) vs. 310 (69.2)	0.713
B vs. non-B, n (%)	28 (18.2) vs. 126 (81.8)	75 (16.7) vs. 373 (83.3)	0.682
AB vs. non-AB, n (%)	11 (7.1) vs. 143 (92.9)	45 (10.0) vs. 403 (90.0)	0.285
RhD+ vs. RhD−, n (%)	134 (87.0) vs. 20 (13.0)	395 (88.2) vs. 53 (11.8)	0.813

* Overall comparison of ABO blood group distribution (4 × 2 chi-square test). Of the 806 patients with epilepsy, 204 (25.3%) with undetermined/unclassifiable genetic etiology were excluded from this subanalysis, leaving 602 patients (154 genetic, 448 non-genetic).

## Data Availability

Data were analyzed in this study but are not publicly available because of institutional and privacy restrictions.

## Supplementary Materials

The following supporting information can be downloaded at https://www.mdpi.com/article/10.3390/jcm15186949/s1, Table S1: Presenting complaints and reasons for evaluation in the control group; Table S2: Goodness-of-fit and multicollinearity diagnostics for the multivariable logistic regression models; Table S3: Post hoc minimum detectable effect size (MDES) and power analysis for ABO/RhD blood group comparisons; Table S4: Unadjusted and Age- and Sex-Adjusted Associations Between ABO/RhD Blood Groups and Genetic Epilepsy Status.
