# Supplementary material for: ABO and RhD Blood Groups in Epilepsy: A Comprehensive Case–Control Analysis
Source: J Clin Med. 2026 Sep 8;15(18):6949. doi: 10.3390/jcm15186949 (PMC13607642; doi:10.3390/jcm15186949)
Supplement: Supplementary file 1 [file jcm-15-06949-s001.zip › jcm-4446353-supplementary.pdf]

Supplementary Table S1. Presenting complaints and reasons for evaluation in the control group

**Presenting complaint or reason for evaluation**	**n**	**%**
Headache	2,626	18.7
Musculoskeletal or pain complaints (neck, back, or hip pain)	2,568	18.3
Sensory complaints (numbness or paresthesia)	1,999	14.2
Dizziness or balance complaints	1,979	14.1
Routine or administrative evaluations*	1,381	9.8
Cognitive complaints	940	6.7
Sleep-related complaints	695	5.0
Syncope or presyncope	688	4.9
Psychiatric or functional complaints	477	3.4
Tremor or movement-related complaints	251	1.8
Other or nonspecific presentations**	223	1.6
Visual complaints	205	1.5
**Total**	**14,032**	**100.0**

Notes: Presenting complaints and reasons for evaluation were obtained from the available hospital records and grouped for descriptive purposes. Categories reflect the primary reason for neurological evaluation rather than systematically adjudicated final neurological diagnoses.

* **Routine or administrative evaluations** included assessments for driver's license, school or dormitory admission, sports participation, course enrollment, medical certification, and decision-making or legal capacity.

** **Other or nonspecific presentations** included infrequent neurological complaints and other reasons for evaluation that did not fit the predefined categories.

Supplementary Table S2. Goodness-of-fit and multicollinearity diagnostics for the multivariable logistic regression models

Model	Nagelkerke R^2	Hosmer–Lemeshow χ^2 (df = 8)	Hosmer–Lemeshow *p*	Maximum VIF
ABO categorical (A, O, B, AB; reference = A)	0.024	15.43	0.051	1.21
Non-O vs O	0.024	16.10	0.041	1.08
Non-A vs A	0.024	15.04	0.058	1.08
Non-B vs B	0.024	19.20	0.014	1.08
Non-AB vs AB	0.024	18.37	0.019	1.08
RhD− vs RhD+	0.024	16.01	0.042	1.08

Abbreviations: VIF, variance inflation factor.

Each row represents a separate multivariable logistic regression model adjusted for age and sex, with epilepsy status as the dependent variable. The reported VIF corresponds to the maximum value among all independent variables (blood group predictor, age, and sex) in each model. All VIF values were below 1.3, indicating no evidence of problematic multicollinearity. Nagelkerke R^2 values were approximately 0.024 across all models, indicating limited overall explanatory capacity. The Hosmer–Lemeshow test reached statistical significance ($p < 0.05$) in several models, indicating potential calibration limitations. These models were used to obtain age- and sex-adjusted estimates of the prespecified associations and were not intended for prediction or classification of epilepsy status.

Supplementary Table S3. Post hoc minimum detectable effect size (MDES) and power analysis for ABO/RhD blood group comparisons

Comparison	Observed OR (95% CI)	n (epilepsy/control)	MDOR at 80% power	Post hoc power for observed effect
non-O vs O	1.05 (0.90–1.22)	806 / 14,032	0.81 / 1.25	9.6%
non-A vs A	0.93 (0.80–1.06)	806 / 14,032	0.82 / 1.23	18.0%
non-B vs B	1.03 (0.85–1.25)	806 / 14,032	0.77 / 1.32	6.2%
non-AB vs AB	1.05 (0.81–1.34)	806 / 14,032	0.72 / 1.46	6.5%
RhD− vs RhD+	1.06 (0.85–1.31)	806 / 14,032	0.73 / 1.33	8.1%
non-O vs O (matched)	—	806 / 3,224	0.79 / 1.28	5.3%
RhD− vs RhD+ (matched)	—	806 / 3,224	0.70 / 1.37	6.3%
ABO overall (χ^2, df = 3)	w = 0.009	N = 14,838	w = 0.027	12.3%
RhD overall (χ^2, df = 1)	w = 0.004	N = 14,838	w = 0.023	7.4%

Abbreviations: OR, odds ratio; CI, confidence interval; MDOR, minimum detectable odds ratio; RhD, Rhesus D.

MDOR represents the smallest odds ratio that the available sample size would detect with 80% statistical power at a two-sided α of 0.05, calculated separately for the protective (lower value) and risk (upper value) directions using the observed control-group prevalence as the reference proportion. w denotes Cohen's effect size for chi-square tests. Post hoc power calculations were performed using the **statsmodels** package (version 0.14) in Python.

Supplementary Table S4. Unadjusted and Age- and Sex-Adjusted Associations Between ABO/RhD Blood Groups and Genetic Epilepsy Status

Comparison	Unadjusted OR (95% CI)	*p*	Age- and sex-adjusted OR (95% CI)	*p*
Non-O vs O	1.08 (0.72–1.61)	0.713	1.07 (0.71–1.62)	0.750
Non-A vs A	0.88 (0.61–1.28)	0.511	0.84 (0.57–1.23)	0.364
Non-B vs B	0.90 (0.56–1.46)	0.682	1.00 (0.61–1.65)	0.993
Non-AB vs AB	1.45 (0.73–2.88)	0.285	1.44 (0.71–2.91)	0.308
RhD− vs RhD+	1.11 (0.64–1.93)	0.704	1.14 (0.64–2.01)	0.660

Abbreviations: OR, odds ratio; CI, confidence interval; RhD, Rhesus D.

Footnote: Odds ratios represent the odds of genetic (vs non-genetic) epilepsy for each blood group comparison. Adjusted odds ratios were obtained from separate multivariable logistic regression models including age and sex as covariates, with genetic epilepsy status (yes/no) as the dependent variable. Of the 806 patients with epilepsy, 204 (25.3%) with undetermined or unclassifiable genetic etiology were excluded from this subanalysis, leaving 602 patients for analysis (154 genetic and 448 non-genetic epilepsy).
